# Cytokine storm and microvascular fate: mechanistic insights into endothelial injury in thrombotic microangiopathies

**DOI:** 10.1097/MS9.0000000000003700

**Published:** 2025-08-08

**Authors:** Emmanuel Ifeanyi Obeagu

**Affiliations:** a Department of Biomedical and Laboratory Science, Africa University, Mutare, Zimbabwe; b Department of Medical Laboratory Science, Kampala International University, Ishaka, Uganda

**Keywords:** cytokines, endothelial dysfunction, inflammation, microvascular thrombosis, thrombotic microangiopathy

## Abstract

Thrombotic microangiopathies (TMAs) encompass a diverse group of syndromes marked by microvascular thrombosis, thrombocytopenia, and organ injury, primarily affecting the kidneys and central nervous system. While the etiologies differ—ranging from genetic mutations to infectious and autoimmune triggers—a unifying pathogenic mechanism is endothelial dysfunction. Recent advances have illuminated the pivotal role of cytokine dysregulation in initiating and sustaining this vascular injury. The release of pro-inflammatory cytokines such as TNF-α, IL-1β, and IL-6 creates a state of sustained endothelial activation that promotes leukocyte adhesion, vascular permeability, and a prothrombotic surface phenotype. In various TMA subtypes, the cytokine response acts as both a trigger and an amplifier of disease progression. In atypical hemolytic uremic syndrome (aHUS), cytokines are upregulated secondary to complement dysregulation, while in thrombotic thrombocytopenic purpura (TTP), inflammation may lower ADAMTS13 activity and potentiate thrombosis. Secondary TMAs, including those associated with autoimmune disease, pregnancy, and transplantation, often exhibit pronounced cytokine profiles that directly correlate with endothelial injury and clinical severity. These overlapping inflammatory signatures underscore the need to view TMAs not only through a hematologic or immunologic lens but also within the context of vascular inflammation.

## Introduction

Thrombotic microangiopathies (TMAs) represent a spectrum of rare but life-threatening disorders characterized by widespread microvascular thrombosis, thrombocytopenia, and microangiopathic hemolytic anemia. Central to their pathophysiology is endothelial injury, which disrupts vascular integrity and triggers platelet aggregation, fibrin deposition, and tissue ischemia. TMAs encompass a heterogeneous group of conditions, including thrombotic thrombocytopenic purpura (TTP), hemolytic uremic syndrome (HUS)—both typical (Shiga toxin-mediated) and atypical (complement-mediated)—and secondary TMAs associated with infections, autoimmune diseases, malignancies, pregnancy, transplantation, and certain medications^[1–4]^. Globally, the incidence of TMAs varies by subtype and population. TTP occurs at an estimated rate of 2–6 cases per million annually, with a female predominance. Shiga toxin-producing *Escherichia coli*-associated HUS (STEC-HUS) remains the most common cause of TMA in children, particularly in areas with poor food or water safety. Atypical HUS (aHUS), though rarer, has been increasingly diagnosed due to improved understanding of complement dysregulation and availability of genetic testing. Secondary TMAs, especially those triggered by malignancies or transplant-associated endothelial injury, may be underrecognized, with their true burden likely underestimated^[5–8]^.

**HIGHLIGHTS**

- Pro-inflammatory cytokines trigger endothelial activation, promoting leukocyte adhesion, thrombosis, and vascular permeability in TMAs.
- Elevated IL-6, TNF-α, and IL-1β levels correlate with disease severity across TMA subtypes.
- Cytokine-mediated endothelial injury amplifies complement activation and propagates microvascular damage.
- Distinct cytokine profiles may aid in diagnosis, prognostication, and treatment personalization in TMAs.
- Targeted cytokine inhibitors offer promising adjunct therapies alongside plasma exchange and complement blockade.

Across TMA subtypes, a unifying pathological feature is endothelial cell activation and damage, which sets the stage for microvascular thrombosis. Among the multiple upstream insults capable of initiating endothelial dysfunction, the cytokine storm—a hyperinflammatory state marked by excessive production of proinflammatory cytokines such as interleukin-6 (IL-6), tumor necrosis factor-alpha (TNF-α), and interleukin-1 beta (IL-1β)—has emerged as a key driver. Cytokine-mediated endothelial activation not only promotes leukocyte adhesion and vascular permeability but also synergizes with complement activation and coagulation pathways, amplifying vascular injury and thrombus formation^[9–12]^. While several reviews have explored the immunologic underpinnings of individual TMA subtypes, a comprehensive synthesis focusing on the interplay between cytokine storms and endothelial fate across the TMA spectrum remains lacking. Such an analysis is essential to inform both pathophysiologic understanding and therapeutic innovation, especially as cytokine-targeted and complement-modulating therapies advance in clinical development. This review aims to elucidate the mechanistic relationship between cytokine storm and endothelial injury in TMAs, emphasizing the differential cytokine profiles among subtypes, including cancer-related and pediatric-onset TMAs. The paper further explored the implications of these mechanisms for current and emerging therapeutic strategies, highlighting opportunities for translational intervention and personalized treatment approaches (Table 1).

**Table 1:** Comparative cytokine profiles across thrombotic microangiopathy (TMA) subtypes

TMA subtype	Major triggers	Key elevated cytokines	Primary immune/inflammatory Pathways	Clinical implications
**Thrombotic thrombocytopenic purpura (TTP)**	ADAMTS13 deficiency (autoimmune or hereditary)	IL-6, TNF-α, IFN-γ, IL-10	Th1/Th17 activation, macrophage polarization	Promotes vWF multimer accumulation, platelet-rich thrombi, systemic inflammation
**Shiga toxin-mediated HUS (STEC-HUS)**	Shiga toxin-producing *E. coli* infection	IL-6, IL-8, TNF-α, MCP-1	Endothelial apoptosis, NF-κB activation	Neutrophil recruitment, glomerular injury, cytokine-mediated hemolysis
**Atypical HUS (aHUS)**	Complement dysregulation (genetic or acquired)	IL-1β, IL-6, C5a, TNF-α	Complement–cytokine crosstalk, inflammasome activation	Amplifies membrane attack complex formation, renal microvascular injury
**Malignancy-associated TMA**	Solid tumors (breast, gastric, etc.), hematologic malignancies	IL-8, VEGF, IL-6, GM-CSF	Tumor–endothelium interaction, angiogenic signaling	VEGF overexpression disrupts endothelial integrity, exacerbating microthrombosis
**Transplant-associated TMA (TA-TMA)**	Hematopoietic stem cell or solid organ transplant	IL-6, IFN-γ, TNF-α, sVCAM-1	Graft-versus-host disease (GVHD)-linked cytokine storm	Endothelial activation with complement amplification, risk of graft loss
**Pregnancy-Related TMA (e.g., HELLP syndrome)**	Preeclampsia, eclampsia, or pregnancy-triggered aHUS	IL-6, TNF-α, IL-1β, sFlt-1	Anti-angiogenic imbalance, inflammasome activation	Endothelial dysfunction in placenta and systemic vasculature; adverse maternal-fetal outcomes
**COVID-19-associated TMA**	SARS-CoV-2 infection	IL-6, IL-1β, IL-10, IFN-γ, CXCL10	Hypercytokinemia, endothelialitis, complement overactivation	Diffuse microvascular thrombosis, multi-organ failure, capillary leak syndrome
**Drug-induced TMA**	Calcineurin inhibitors, chemotherapy agents	TNF-α, IL-6, IL-1β	Oxidative stress, drug-mediated endothelial injury	Dose-dependent endothelial apoptosis and thrombotic changes
**Pediatric TMA (various etiologies)**	Congenital defects, infection, complement mutation	IL-6, IL-8, IL-10, TNF-α	Immature immune response, cytokine overexpression	Age-specific cytokine patterns may lead to rapid clinical deterioration

### Aim

This review aims to critically examine the mechanistic role of cytokine storms in endothelial injury across TMA subtypes. Specifically, it explores differential cytokine profiles—including those in cancer-related and pediatric-onset TMAs—while highlighting how these inflammatory patterns inform the pathogenesis, clinical presentation, and therapeutic strategies. By integrating current evidence, the review seeks to identify translational opportunities for cytokine- and complement-targeted interventions in the management of TMAs.

## Gaps in knowledge

Despite recent advances in understanding the immunopathogenesis of TMAs, several critical gaps remain. Firstly, the precise temporal sequence of cytokine elevation and endothelial injury is not well established. Most studies offer cross-sectional data without capturing the dynamic interplay between inflammatory mediators and vascular response. Secondly, the role of lesser-known cytokines (e.g., IL-8, VEGF, MCP-1) and endothelial-derived exosomes in initiating or sustaining vascular injury is underexplored. Thirdly, heterogeneity across TMA subtypes—particularly in malignancy-associated or pediatric TMAs—has not been systematically characterized in terms of cytokine signatures or therapeutic responsiveness. Lastly, there is insufficient stratification of patients based on molecular or immunological profiles, which limits the development of precision-based interventions.

## Limitations of current studies

A major limitation in the current body of literature is the predominance of retrospective observational studies, small cohorts, and case series, which limit generalizability and preclude causal inference. The absence of standardized diagnostic criteria for cytokine storms within TMA contexts contributes to inconsistent reporting. Moreover, many investigations rely solely on peripheral blood cytokine levels, which may not accurately reflect local endothelial environments in affected microvascular beds. Clinical trials evaluating cytokine-targeted therapies in TMAs are scarce, and when available, they often lack robust control arms or long-term outcome data. Additionally, most studies fail to account for age-specific immune responses, especially in pediatric populations where endothelial physiology and immune activation differ significantly from adults.

## Cytokine-mediated endothelial activation

Endothelial cells form the critical interface between circulating blood and the vessel wall, maintaining vascular homeostasis by regulating permeability, vascular tone, and hemostasis. Under physiological conditions, the endothelium exhibits an anti-thrombotic, anti-inflammatory phenotype, producing factors such as nitric oxide and prostacyclin that inhibit platelet aggregation and leukocyte adhesion. However, in the setting of TMAs, the endothelium undergoes a dramatic phenotypic transformation triggered largely by cytokine-mediated activation^[2,13,14]^. Pro-inflammatory cytokines, particularly TNF-α, IL-1β, and IL-6, play a central role in this process. Upon exposure to these cytokines, endothelial cells upregulate adhesion molecules including intercellular adhesion molecule-1 (ICAM-1), vascular cell adhesion molecule-1 (VCAM-1), and E-selectin. This enhanced expression facilitates the adhesion and transmigration of leukocytes, contributing to vascular inflammation and local tissue injury. Furthermore, cytokine stimulation promotes the secretion of von Willebrand factor (vWF) from Weibel-Palade bodies, which increases platelet tethering and aggregation on the endothelial surface, predisposing to microthrombus formation^[15,16]^.

**Figure 1.**
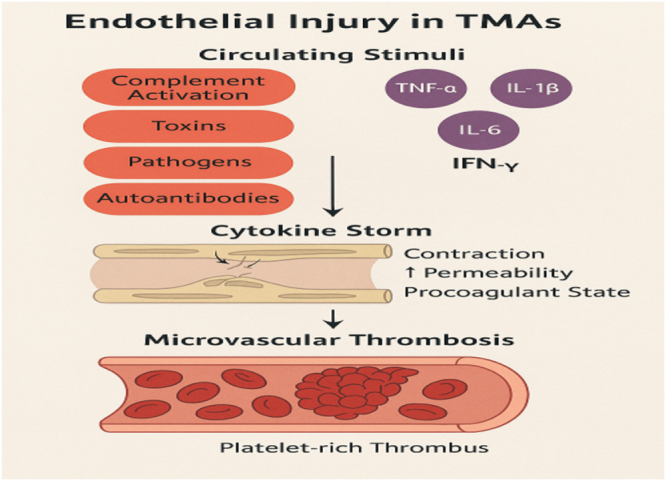
Mechanistic pathway of endothelial injury in TMAs.

Simultaneously, cytokines modulate the balance between procoagulant and anticoagulant factors on the endothelium. TNF-α and IL-1β induce tissue factor expression, initiating the extrinsic coagulation cascade, while downregulating thrombomodulin and the endothelial protein C receptor, which are key components of the anticoagulant pathway. This shift favors fibrin deposition and platelet-rich thrombus formation within the microvasculature. In parallel, cytokines reduce endothelial nitric oxide synthase (eNOS) activity, leading to decreased nitric oxide production. The resultant endothelial dysfunction promotes vasoconstriction, platelet activation, and further inflammatory cell recruitment, creating a vicious cycle of vascular injury^[17–19]^. Beyond these direct effects, cytokines also influence endothelial permeability. IL-6 and TNF-α disrupt tight junction proteins, such as occludins and claudins, increasing vascular leakage and edema. This heightened permeability not only exacerbates tissue ischemia but also allows plasma proteins and inflammatory mediators to extravasate, amplifying local inflammation. Moreover, sustained cytokine exposure impairs endothelial repair mechanisms by inhibiting endothelial progenitor cell mobilization and function, thereby compromising vascular regeneration and perpetuating microvascular damage (Fig. 1)^[20–22]^.

## Cytokine profiles across TMA subtypes

TMAs encompass a spectrum of disorders with distinct etiologies, yet many share overlapping cytokine-mediated mechanisms that contribute to endothelial injury and microvascular thrombosis. Investigating cytokine profiles in different TMA subtypes provides valuable insights into their pathogenesis and reveals potential targets for tailored therapeutic interventions^[23,24]^. In **aHUS**, a disorder primarily driven by dysregulation of the alternative complement pathway, cytokine release plays an amplifying role in endothelial injury. Complement activation products, especially C5a, stimulate endothelial cells and immune cells to produce pro-inflammatory cytokines such as TNF-α, IL-6, and interleukin-8 (IL-8). These cytokines enhance leukocyte recruitment and promote a prothrombotic endothelial phenotype, exacerbating vascular damage. Studies have demonstrated elevated serum IL-6 and TNF-α levels during active disease phases, correlating with disease severity and renal impairment. This cytokine milieu creates a feedback loop that intensifies complement activation and endothelial dysfunction, driving microvascular thrombosis^[15,25,26]^.

In **TTP**, the primary pathogenic mechanism involves a severe deficiency of ADAMTS13, the metalloprotease responsible for cleaving ultra-large vWF multimers. However, inflammatory cytokines also significantly influence disease expression and progression. Elevated levels of IL-6, TNF-α, and interferon-gamma (IFN-γ) have been observed during acute TTP episodes. These cytokines contribute to endothelial activation and promote the release of ultra-large vWF multimers from endothelial Weibel-Palade bodies. Additionally, inflammatory mediators may impair ADAMTS13 synthesis or function, further propagating microvascular thrombosis. Cytokine elevations often parallel clinical exacerbations and may serve as markers of disease activity^[2,27,28]^. **Secondary TMAs**, occurring in association with conditions such as systemic lupus erythematosus, pregnancy-related complications (e.g., HELLP syndrome), infections, malignancies, or drug toxicity, frequently exhibit distinct cytokine signatures reflective of their underlying triggers. For instance, in pregnancy-associated TMAs, elevated TNF-α, IL-6, and interferon-alpha (IFN-α) levels contribute to placental endothelial dysfunction, promoting microangiopathy. Autoimmune-related TMAs show increased production of pro-inflammatory cytokines such as IL-1β and IL-18, which drive systemic inflammation and endothelial injury. Similarly, in transplant-associated TMA, cytokines like interferons and tumor necrosis factor are induced by conditioning regimens and graft-versus-host responses, exacerbating vascular damage^[29–31]^.

## Therapeutic implications

Understanding the intertwined roles of cytokines and endothelial dysfunction in TMAs has laid the groundwork for novel therapeutic strategies. Traditional treatment modalities, such as plasma exchange and supportive care, remain vital, particularly in TTP and aHUS. However, these approaches often fail to address the underlying immunopathogenic mechanisms—especially the synergistic effect of cytokine storms and complement overactivation. This has sparked interest in targeted immunotherapies, some of which are now entering clinical practice or undergoing evaluation in early-phase trials[32].

## Emerging dual-targeting therapies: complement–cytokine blockade

Recent insights suggest that neither cytokine storm nor complement activation occurs in isolation; instead, they reinforce each other to propagate endothelial injury. This has led to the conceptualization of dual-targeting approaches aimed at simultaneously dampening both axes of inflammation. One promising example is the concurrent inhibition of IL-6 (using agents like tocilizumab or satralizumab) and complement components (such as C5 with eculizumab or ravulizumab). This dual blockade may be particularly beneficial in secondary TMAs like COVID-19-associated or transplant-related cases, where hypercytokinemia and complement dysregulation coexist. Preclinical studies and small cohort trials suggest improved control of inflammation, endothelial preservation, and reduction in thrombotic burden when this combination strategy is employed^[33,34]^.

## Investigational agents and their potential in clinical practice

A number of investigational therapies targeting novel inflammatory pathways are currently under evaluation. These include:

- **Narsoplimab**, a MASP-2 inhibitor, which targets the lectin pathway of complement activation and shows promise in transplant-associated TMA.
- **Vilobelimab**, a C5a inhibitor, which is under investigation in inflammatory microangiopathies, particularly those with neutrophil-dominant pathology.
- **Anakinra**, an IL-1 receptor antagonist, has been repurposed from rheumatologic settings for use in TMA-like syndromes with prominent IL-1β upregulation.
- **BTK inhibitors** (e.g., rilzabrutinib) and JAK inhibitors (e.g., ruxolitinib) are also under exploration for their capacity to attenuate both innate immune activation and downstream cytokine production, offering a broader scope of control over inflammatory microenvironments[2].

While many of these agents remain in early clinical development or off-label use, they represent a shift toward precision immunotherapy in TMA management. Their integration into practice will depend on future validation through randomized trials and biomarker-driven patient selection.

## Biologics vs. small molecules: comparative therapeutic landscape

Biologics such as monoclonal antibodies (e.g., eculizumab, tocilizumab, anakinra) offer high specificity and prolonged action, making them ideal for chronic or severe TMAs. However, their high cost, parenteral administration, and potential for immune-related adverse events are limiting factors. In contrast, small molecules—including JAK inhibitors and BTK inhibitors—tend to have oral bioavailability, broader immunomodulatory effects, and shorter half-lives, allowing for greater flexibility in dose titration and discontinuation in acute settings^[35,36]^. That said, small molecules often lack the selectivity of biologics, which could lead to off-target effects or increased immunosuppression. Moreover, drug-drug interactions, especially in polypharmacy contexts like transplant or oncology settings, pose a challenge. Thus, an optimal therapeutic strategy may involve phased or combinatorial use of both classes, depending on disease severity, patient comorbidities, and molecular profiling.

## Differential cytokine profiles among TMA subtypes: clinical and pathophysiological perspectives

While TMAs share common histopathological features—namely endothelial injury, platelet-rich microthrombi, and microangiopathic hemolytic anemia—the underlying immunologic landscapes differ significantly across subtypes. These differences are especially pronounced in the profiles of circulating cytokines, which reflect the triggering mechanisms and immunopathogenic processes driving endothelial damage. Recognizing these distinctions is crucial for improving diagnostic specificity, risk stratification, and therapeutic targeting^[37,38]^.

## TTP vs. complement-mediated aHUS

In TTP, cytokine elevation typically results from immune dysregulation associated with autoantibodies against ADAMTS13. The cytokine profile is marked by elevated IL-6, TNF-α, and IFN-γ, with notable increases in IL-10, suggesting a counter-regulatory anti-inflammatory response. These cytokines are associated with macrophage activation and systemic inflammation, which may further inhibit ADAMTS13 activity and exacerbate microvascular thrombosis. In contrast, aHUS, driven by uncontrolled complement activation, presents a cytokine environment dominated by IL-1β, IL-6, C5a, and TNF-α. The interplay between the complement system and proinflammatory cytokines (particularly IL-1β and C5a) amplifies endothelial injury through inflammasome activation, membrane attack complex (MAC) formation, and enhanced leukocyte recruitment. Unlike TTP, IL-10 levels in aHUS are typically lower, indicating a skewed inflammatory balance^[39]^.

## Cancer-related TMA

Malignancy-associated TMA represents a distinct entity with both mechanical and biochemical drivers of vascular injury. Cytokine profiles in cancer-related TMA often include elevated IL-8, VEGF, IL-6, and GM-CSF. These mediators are secreted not only by immune cells but also directly by tumor cells and the tumor-associated stroma. VEGF, in particular, plays a dual role—promoting abnormal angiogenesis while increasing vascular permeability and disrupting endothelial integrity. Elevated IL-8 and GM-CSF contribute to neutrophil recruitment and activation, fostering a prothrombotic state. Additionally, some chemotherapy agents (e.g., mitomycin C) may induce cytokine release from injured endothelial cells, compounding the inflammatory milieu. Clinically, cancer-related TMAs tend to be less responsive to plasma exchange, and prognosis often hinges on controlling the underlying malignancy. Recognition of these unique cytokine patterns may help distinguish cancer-TMA from primary forms and guide adjunctive anti-cytokine or anti-angiogenic therapies^[40,41]^.

## Pediatric-onset TMA

In pediatric populations, TMA manifestations can differ significantly due to age-related immune maturation and variable genetic predispositions. Typical HUS, often caused by Shiga toxin-producing *Escherichia coli*, is the most frequent TMA subtype in children. It is characterized by a surge in IL-6, IL-8, TNF-α, and MCP-1, with IL-8 playing a central role in neutrophil-mediated glomerular injury. The Shiga toxin itself acts synergistically with cytokines to induce endothelial apoptosis and microvascular thrombosis. In pediatric aHUS, complement mutations may present early in life with disproportionately high levels of IL-1β and C5a, which are linked to aggressive clinical courses. Importantly, children often exhibit a more robust innate immune response than adults, potentially exacerbating the cytokine storm. Moreover, age-specific pharmacokinetics and immune responses necessitate tailored therapeutic approaches, particularly in the use of complement or cytokine inhibitors^[42]^.

### Clinical relevance of cytokine profiling

Differentiating TMA subtypes based on cytokine signatures offers several potential clinical advantages:

- Improved diagnostic accuracy, particularly in atypical or overlapping presentations (e.g., cancer vs. aHUS).

- Therapeutic targeting, where cytokine profiles may predict responsiveness to agents such as tocilizumab (IL-6 blockade), eculizumab (C5 inhibition), or anakinra (IL-1 receptor antagonism).
- Prognostic stratification, as specific cytokine patterns (e.g., sustained IL-8 or VEGF in cancer-TMA) may be associated with worse outcomes or treatment resistance^[42]^.

## Future directions

To advance the field, future research must focus on several key areas. Prospective, multicenter studies employing longitudinal cytokine profiling and endothelial biomarkers are needed to elucidate causal pathways and identify early diagnostic markers. Single-cell transcriptomic and proteomic approaches may reveal cell-specific cytokine responses and endothelial heterogeneity across TMA subtypes. There is also a pressing need for randomized controlled trials evaluating the efficacy and safety of cytokine- and complement-targeted therapies, including dual-targeting strategies that inhibit both arms of the inflammatory cascade. Development of predictive models incorporating cytokine panels, endothelial injury markers, and clinical parameters could facilitate risk stratification and personalized treatment. Finally, collaborative efforts between immunologists, hematologists, and vascular biologists will be crucial in translating mechanistic insights into effective therapeutic interventions^[43–45]^.

## Conclusion

Thrombotic microangiopathies represent a complex group of disorders in which cytokine-mediated endothelial injury plays a central role in driving microvascular thrombosis and organ dysfunction. The intricate interplay between proinflammatory cytokines and complement activation varies across TMA subtypes, with distinct cytokine signatures observed in cancer-related and pediatric-onset forms. Understanding these nuanced differences is critical for improving diagnostic accuracy and guiding personalized therapeutic approaches. Emerging dual-targeting therapies that concurrently modulate the complement system and cytokine signaling pathways hold promise for more effective management of TMAs. However, significant gaps remain in our understanding of the optimal timing, patient selection, and long-term outcomes of these interventions. Future research should prioritize well-designed randomized controlled trials and mechanistic studies to clarify the therapeutic potential of cytokine blockade and complement inhibition.

## Data Availability

Not applicable as this a narrative review.

## Research registration unique identifying number (UIN)

Not applicable as this a narrative review.
